# *Chlamydia psittaci*–related pleuro-myocarditis

**DOI:** 10.1016/j.bjid.2024.103739

**Published:** 2024-04-26

**Authors:** Nour Sreiri, Yousri Ben Abdallah, Nabil Belfeki, Timothée Klopfenstein, Souheil Zayet

**Affiliations:** a Nord Franche-Comté Hospital, Infectious Diseases Department, Trévenans, France; b Nord Franche-Comté Hospital, Pneumology Department, Trévenans, France; c Groupe Hospitalier Sud Ile de France, Internal Medicine and Clinical Immunology Department, Melun, France

**Keywords:** *Chlamydia psittaci*, Community-acquired pneumonia, Acute myocarditis, Polymerase chain reaction

## Abstract

*Chlamydia psittaci* ‒ related community-acquired pneumonia associated to acute myocarditis was diagnosed in a young man with no medical history, and a professional exposition to birds. The diagnosis was confirmed with positive specific polymerase chain reaction in bronchoalveolar lavage. The patient was treated with spiramycin for two weeks with anti-inflammatory treatment for myocarditis for three months. Clinical and biological improvement was rapidly observed followed by normalization of electrocardiogram and chest CT scan. No relapse was reported for over a two-year follow-up.

## Introduction

Psittacosis, also commonly known as the avian chlamydiosis, is a rare infection caused by an intracellular Gram-negative coccoid organism called *Chlamydia psittaci*. It is transmitted by inhalation of bird's excretions. It infects birds mostly, such as parrots, parakeets, pigeons and particularly mulard ducks in France (intended for the production of ‘foie gras’). Studies show that the carriage of *C. psittaci* in this sector is very frequent. It is less frequent in turkey breeders or in slaughterhouses. Fifty percent of birds are seroprevalent with an inter species variation.^1^ Patients with history exposure to birds have an increased risk for developing the disease.

About one hundred human cases were reported in France up until 2015 according to the national disease control institute.^1^

We have reported a scare case of *C. psittaci* pneumonia complicated with myocarditis.

## Case report

**Fig. 1:**
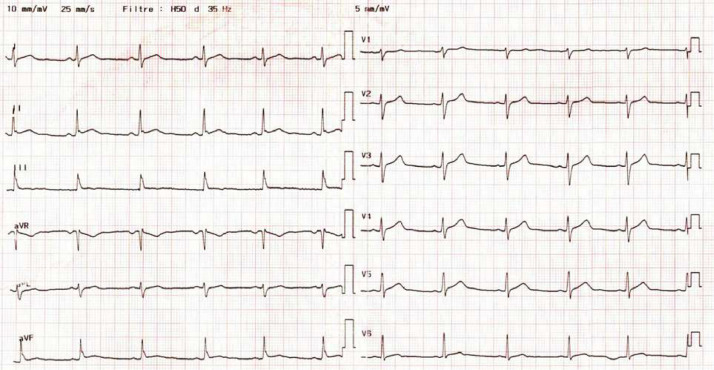
Electrocardiogram showing early repolarisation.

**Fig. 2:**
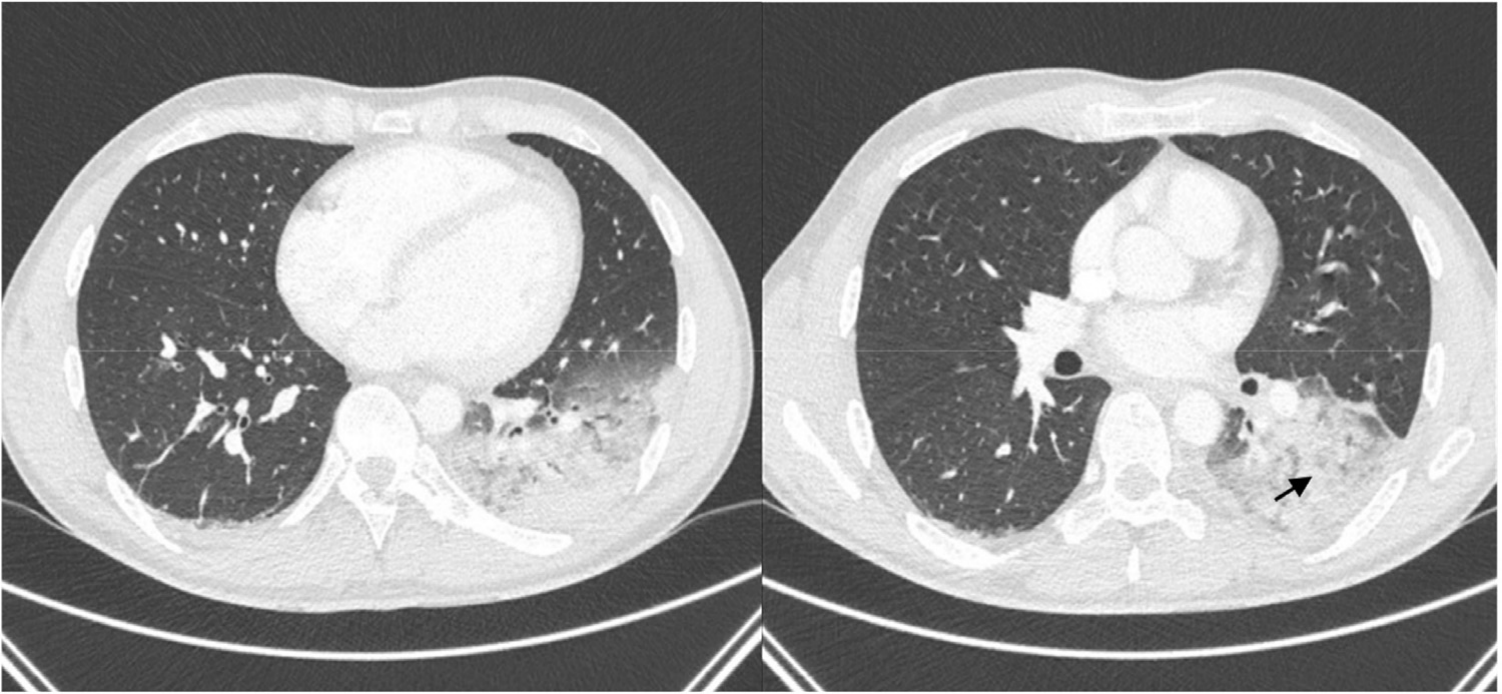
Chest computed tomography scan showed subpleural ground-glass opacities (black arrow) with a basal distribution in the left lower lobe and alveolar consolidations.

A 30-year-old man, with no past medical history, presented initially to the emergency room for a three-day history of chest constrictive pain with productive cough, headaches and diarrhea associated with fever at 39 °C. He had not travelled abroad during the last months. He was a non-smoker and a non-drug user but did drink alcohol occasionally. He also has dogs, a rabbit, and a guinea pig at home. At admission, physical examination confirmed a high temperature of 39 °C, revealed a normal blood pressure of 135/80 mmHg, normal pulse rate of 90 bpm and right basal sibilants on pulmonary auscultation. The respiratory rate was normal, and the oxygen saturation was 96 % on ambient air. Laboratory tests showed mild leukolymphopenia (leukocytes 3240 µL [normal range 4000–10,000 µL]), lymphocytes 780 µL (normal range 1500–4000 µL), with a normal platelet count (280 000 µL [normal range 150,000–400,000 µL]). We also noted an increased high-sensitivity Troponin-T (hsTnT) level at 2442 ng/L (normal range < 34 ng/L) and a high level of C-Reactive-Protein (CRP) at 177 mg/L (normal range < 5 mg/L). Serum electrolytes, renal and liver parameters were in normal range. The electrocardiogram showed early repolarisation (Fig. 1). Thoracic Computed Tomography (CT) scan showed alveolar consolidations in the left lower lobe associated with subpleural ground‐glass opacities with a basal distribution (Fig. 2). Transthoracic echocardiography revealed a preserved ejection fraction of 65 %, with normal wall motions with no valvular dysfunction, normal pulmonary pressure and no pericardial effusion, however the right ventricle was echogenic.

Clinical, biological and imaging findings (thoracic CT scan and echocardiographic presentations) summed the hypothesis of Community-Acquired Pneumonia (CAP) associated with an acute myocarditis. The patient underwent bronchoscopy with Bronchoalveolar Lavage (BAL) which collected an inflammatory sterile fluid (240 white cells count, 54 % alveolar macrophages). The BAL microscopic examination for Acid-Fast-Bacilli (AFB) was negative, as well as culture. BAL cytology did not show malignant cells.

Antimicrobial empiric treatment was started Simultaneous Intravenous (IV) administration of amoxicillin (1 g t.i.d) and spiramyci 1.5 MUI t.i.d associated to colchicine 0.5 mg q.i.d and oral acetylsalicylic acid 1 g t.i.d as the myocarditis treatment. Legionella and pneumococcal urinary antigen tests were negative. Repeated peripheral blood cultures were also negative. Further investigations including QuantiFERON-TB Gold test and human immunodeficiency virus serology were negative. *Mycoplasma pneumoniae, C. pneumoniae, Coxiella burnetti* and first *C. psittaci* serology's (performed seven days after symptoms onset) were negative. Detection of respiratory viruses such as SARS-CoV-2, Influenza A/B and syncytial respiratory virus were negative in nasopharyngeal swabs. Biomolecular detection in the respiratory fluid (BAL) was positive for *C. psittaci* (using real time Polymerase Chain Reaction [r-t-PCR] TaqMan probe, LightCycler and rDNA 23 s) confirming the diagnosis of psittacosis with pulmonary and cardiac features. Anamnestic investigations concluded that our patient works as a carpenter and was manipulating a bird's nest frame six hours before the onset of the symptoms. The patient started to improve after two days of starting antimicrobial drugs; he no longer had fever or chest pain. Laboratory tests showed normal blood cell count (leukocytes 5480 µL, lymphocytes 1430 µL), and decreased CRP at 23 mg/L (day 5); hsTnT levels also decreased to 12.9 ng/L. Amoxicillin was stopped and spiramycin was conducted for a total of 14 days without adverse effects. Follow-up *C. psittaci* serology 21 days after onset of symptoms was positive (on immunoglobulin IgM and IgG). Follow-up chest CT scan, performed two months later, showed a disappearance of pulmonary consolidations with no other abnormalities. Colchicine and oral acetylsalicylic acid were continued for three months. Electrocardiogram following treatment course was normal. Two years after the patient has never had relapse.

## Discussion

This clinical case report illustrates a scarce and atypical form of psittacosis presented as inaugural acute myocarditis associated with a CAP lobar pneumonia in a young male patient. Psittacosis most likely causes pneumonia with several respiratory features. Cardiac presentations are described in chlamydial infections but few of them imputed to *C. psittaci*. To the best of our knowledge, only three cases of myocardial psittacosis have been reported in the last century of medical literature.^2^, ^3^, ^4^ Other cardiac involvements such as pericarditis and endocarditis have scarcely been reported.^5^ The diagnosis of myocarditis in our patient was based on chest pain associated with a high level of cardiac biomarkers and non-specific repolarisation changes. The early repolarisation pattern was recently described in myocarditis but not associated with a worse prognosis and it was reversible over time along with the normalization of the cardiac biomarkers^6^ like what we experienced with our patient. One of our major limitations in this current paper is that we did not perform a cardiac magnetic resonance imaging. Physiopathology remains not well known. In a study conducted by G. Wang et al.^7^ which inoculated cardiomyocytes of neonate rats with *chlamydial* spp. concluded that this bacterium can infect and replicate in myocytes causing significant damage to the cells, as evidenced by an increased lactate dehydrogenase release, reactive oxygen species production and a reduced adenosine triphosphate level without nuclear apoptosis. The disease is likely to be under-diagnosed since patients have non-specific presentations consisting in influenza-like illnesses with a long term fever.^8^ Sometimes, according to the infected organ, psittacosis may present as acute hepatitis, meningitis or encephalitis^9^ or gastroenteritis^8^ making the diagnosis even more complicated especially when clinical features are exclusively non-respiratory symptoms. That is why anamnestic investigations by searching for contact history with birds are essential even if it is not always reported. The prevalence of CAP caused by *C. psittaci* ranges from 1 %‒6.7 % of all pneumonia causes.^10^ In more than half cases,^8^ a single lower lobe is involved, with rare cases of associated pleural effusions. Radiological findings for our patient were consistent with literature. However, less frequent imaging findings were reported such as extended bilateral pneumonia or organizing monolobar pneumonia.^8^^,^^11^ It is important to rule out other intracellular pneumonia causes such as legionellosis, like we did, since clinical presentation can be very similar.

The diagnosis is based on serology and more recently on molecular biology in respiratory samples. Serology is usually negative the first ten days after symptoms onset explaining our first serology results, which was probably performed early (on day seven). It also lacks of specificity since there is many cross-reactivity with other chlamydial bacteria. The serology is best interpreted with a two-week follow-up control to confirm seroconversion. Currently, detection of *C. psittaci* using r-t-PCR, is faster and more sensitive than serologic tests. According to the affected organ, PCR can be used on respiratory specimens, blood,^12^ stool samples, tissue biopsies in some atypical cases^8^^,^^13^ and even cerebrospinal fluid in neurological features.^9^ In a recent study that collected 54 human specimens of *C. psittaci* by using r-t-PCR,^13^ the authors concluded that *C. psittaci* were more frequently detected in lower respiratory specimens (59 % [10 of 17]) and stool (four of five) than in upper respiratory specimens (7 % [2 of 28]). Cycle threshold (Ct) values suggested bacterial load was higher in lower respiratory specimens than in nasopharyngeal swabs. In our case, no nasopharyngeal swab was tested but the analysis of BAL confirmed the diagnosis of psittacosis.

The treatment is similar to other CAP induced by intracellular pathogens, especially caused by *C. pneumoniae*. Doxycycline is the preferred regimen for these infections. Some authors experienced success in severe pneumonia using new cycline-derived drugs such as tigecycline^14^ and omadacycline^15^ after usual regimens proved unsuccessful. Macrolides-based regimens have proven to be also effective. In Branley et al.^8^ study, 40 % of patients were treated by macrolides (roxithromycin, erythromycin, josamycin) with favourable outcomes. Macrolides are known to be excellent antibiotics against intracellular pathogens by enhancement of phagocytosis, inhibition of oxidant production and modulation of cytokine production.^16^ Even though quinolones are considered as alternatives, many case series treated by moxifloxacin reported failure.^14^

Not all cardiac forms of psittacosis are treated the same. In myocarditis, no antibiotic was particularly preferred or more effective. Endocarditis,^5^ on the other hand, was likely treated by doxycycline for a longer period than pulmonary forms exceeding sometimes four months but guidelines tend to not be very well codified . Some authors reported positive blood cultures to *C. psittaci* during endocarditis. This cardiac form is associated with poor prognosis probably due to delay in diagnosis of several weeks and to the fact that surgery is frequently required. Given the known cardiac toxicity of moxifloxacin and macrolides, would it be wise to choose these antibiotics in treating psittacosis with cardiac involvement or would it be preferable to use doxycycline? In our case, no adverse effects were noted nor an outcome worsening using spiramycin. Maybe because this macrolide is not the most cardiotoxic unlike erythromycin, clarithromycin then azithromycin. Along with antibiotics, treatment should also cover myocarditis using anti-inflammatory agents such as colchicine and acetylsalicylic acid.

Contact with birds is only obtained after repeated questioning; either the physician does not suspect psittacosis until first treatment failure, or the patient does not remember a past history of quick contact with birds unless it is a professional exposure.

Though, repeated, and targeted questioning along with atypical presentation can make the diagnosis a bit tricky, psittacosis remains in most cases associated with good prognosis. In some studies reporting mild infections, four patients did even improve spontaneously without antibiotics.^8^

## Conclusion

Psittacosis is an ornithosis usually presenting as pneumonia. However, rare presentations with cardiac involvement are described. Apart from endocarditis, it is usually associated with good prognosis, which can be improved by early diagnostic by asking about bird's exposition. This case highlights also the importance of performing PCR *C. psittaci* assay (especially in lower, respiratory samples) for early diagnosis, pending serology's positivity. Treatment is mostly based on doxycycline, but further studies are necessary to determine the best regimen in case of atypical feature such as cardiac psittacosis.

## Ethics statement

NA.

## Data availability

Data available on request due to privacy restrictions. The data presented in this case study are available on request from the corresponding author.

## Statement and informed consent

NA.

## Funding

This research did not receive any specific grant from funding agencies in the public, commercial, or not-for-profit sectors.

## CRediT authorship contribution statement

**Nour Sreiri:** Conceptualization, Data curation, Formal analysis, Investigation, Methodology, Software, Validation, Writing – original draft. **Yousri Ben Abdallah:** Conceptualization, Data curation, Formal analysis, Project administration, Resources, Software. **Nabil Belfeki:** Validation. **Timothée Klopfenstein:** Supervision, Validation, Visualization, Writing – review & editing. **Souheil Zayet:** Conceptualization, Data curation, Formal analysis, Funding acquisition, Investigation, Methodology, Project administration, Resources, Supervision, Validation, Visualization, Writing – original draft, Writing – review & editing.

## Conflicts of interest

The authors declare no conflicts of interest.
